# Unraveling Lymphatic Filariasis in an Old Man: A Case Report

**DOI:** 10.7759/cureus.58167

**Published:** 2024-04-13

**Authors:** Alisha Handa, Abhay Gaidhane, Sonali Choudhari

**Affiliations:** 1 Community Medicine, Jawaharlal Nehru Medical College, Datta Meghe Institute of Higher Education and Research, Wardha, IND

**Keywords:** elephantiasis, mass drug administration, preventive medicine, old man, lymphatic filariasis

## Abstract

Lymphatic filariasis, caused by filarial worms such as *Wuchereria bancrofti*, *Brugia malayi*, and *Brugia timori*, represents a significant public health burden in endemic regions. The disease primarily affects the lymphatic system, leading to lymphatic dysfunction and chronic morbidity. This abstract provides a comprehensive overview of lymphatic filariasis, including its transmission dynamics, pathogenesis, clinical manifestations, diagnostic approaches, and treatment options. Special attention is given to the socioeconomic impact of the disease and the challenges associated with its control and elimination. The patient in this particular case is a 58-year-old man who had lower limb swelling and pain, characteristic of chronic lymphatic obstruction. Additionally, the swelling tends to worsen during the evening hours often resulting in difficulty in walking and discomfort. Lymphatic filariasis was diagnosed based on clinical presentation.

## Introduction

Lymphatic filariasis (LF), often referred to as elephantiasis, is a neglected tropical disease characterized by the transmission of filarial parasites to humans via mosquitoes. Typically contracted during childhood, the infection silently impacts the lymphatic system, causing internal damage. In 2021, 882.5 million individuals across 44 countries inhabited regions necessitating preventive chemotherapy to halt infection transmission. The initial global estimate revealed that 25 million men were afflicted with hydrocele, while over 15 million individuals suffered from lymphoedema due to LF. Presently, at least 36 million people continue to endure the chronic manifestations of these diseases [1]. LF is triggered by the presence of parasites categorized as nematodes (roundworms) belonging to the Filariodidea family. These parasites are transmitted through the bites of mosquitoes carrying the infection. The larvae, transmitted by mosquitoes, are deposited onto the skin, providing an entry point into the body. Subsequently, these larvae migrate to the lymphatic vessels, where they mature into adult worms, perpetuating the cycle of transmission [2]. Individuals affected with the disease may experience lymphoedema and elephantiasis, with men also encountering scrotal swelling known as hydrocele. LF stands as a prominent cause of permanent disability on a global scale. Various species of mosquitoes can serve as vectors transmitting the parasite, with the specific type varying by geographic region. Individuals residing in tropical and subtropical regions where the disease is prevalent face the highest risk of infection. In Africa, the predominant vector is *Anopheles*, while in the Americas, the transmission is primarily facilitated by *Culex quinquefasciatus*. In the Pacific and Asia, *Aedes* and *Mansonia* mosquitoes are responsible for transmitting the infection. [3]. Contrary to many other helminth infections prevalent in tropical regions, the prevalence of infection and resulting disease burden in LF predominantly manifests during adulthood rather than childhood or infancy. This heightened impact on older age demographics stems from the infection load being influenced by the duration and intensity of exposure to the infectious load being influenced by the duration and intensity of exposure to the infectious stages of the organisms, which are unable to reproduce within the mammalian host [4]. LF is an epidemic in 73 countries and affects approximately 120 million population [5]. The morbidity with LF presents itself through visibly distressing and chronically persistent symptoms, such as lymphoedema (including acute dermatolymphangioadenitis (ADLA) and elephantiasis) and male urogenital issues (like hydrocele and lymph scrotum). ADLA episodes are marked by bacterial infections, leading to considerable pain and fever, often recurring in phases. Additionally, there are less frequently observed symptoms such as breast lymphoedema, vulva swelling, and complications related to rheumatic and respiratory issues [6]. Repeated secondary bacterial infections accelerate the advancement of lymphoedema towards the more severe stage commonly referred to as elephantiasis [7]. Internationally, LF ranks as the second most prevalent cause of chronic disability [8]. The illness places significant economic and psychosocial burdens on individuals affected by it, as well as on their caregivers and families [9]. Global concerns arise from the notion that overall living conditions and mobility contribute to heightened susceptibility to elevated microfilaremia levels in certain populations compared to others [5]. The development of an effective long-term prophylactic strategy, which includes the implementation of a vaccine, is crucial for limiting and eventually eradicating LF in endemic regions [10]. Filariasis treatment typically involves a combination of drugs such as diethylcarbamazine, ivermectin, and albendazole. These medications are administered together to diminish microfilariae levels in the bloodstream and in the skin (onchocerciasis) [11].

## Case presentation

**Figure 1:**
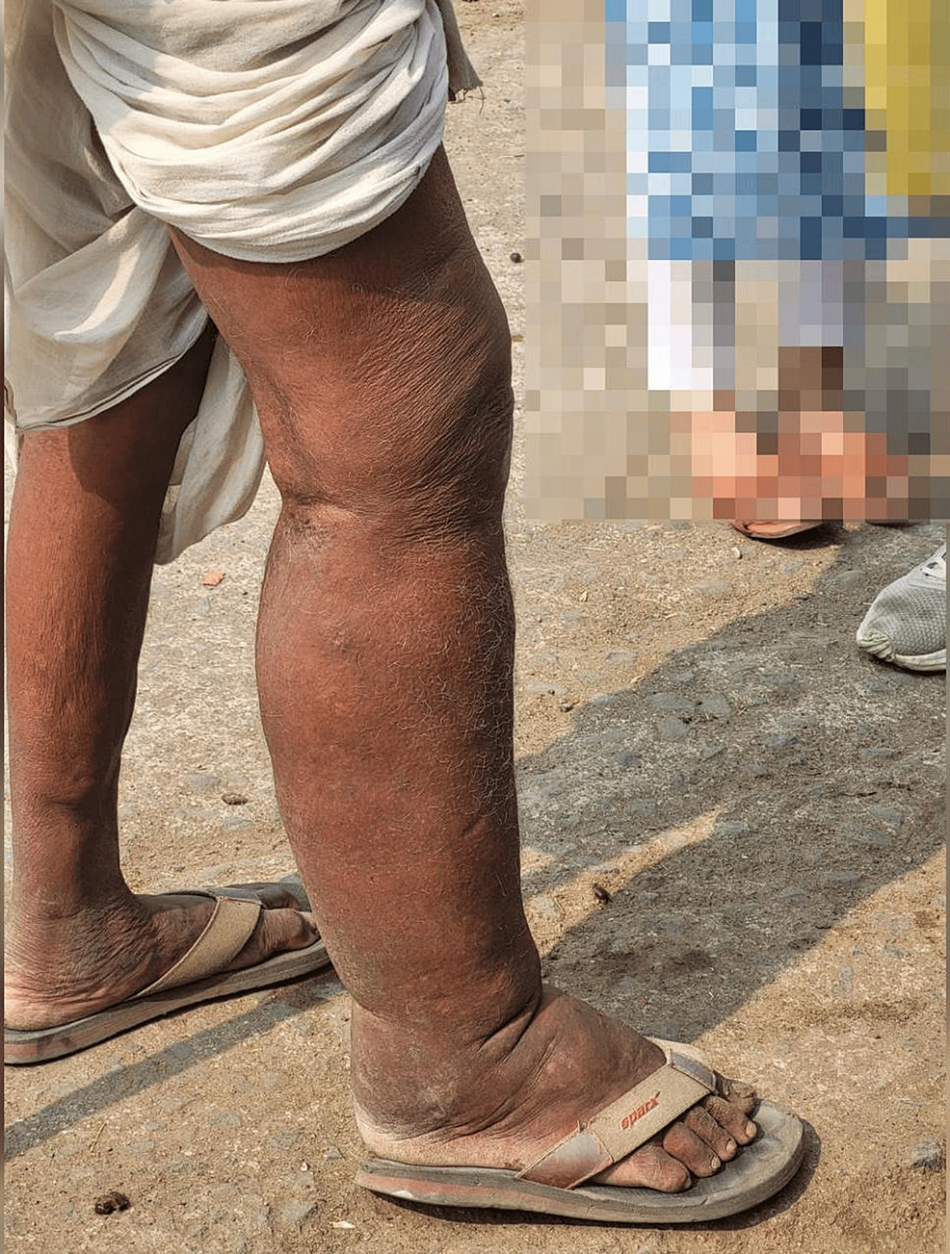
Asymmetric lower limb swelling: impact of lymphatic filariasis

A 58-year-old man residing in a rural village in an endemic area presents with chronic swelling of his lower limb, recurrent fevers, and severe discomfort. On examination, he exhibited signs of lymphoedema (Figure 1). He reported a history of long-term exposure to mosquito bites. During the acute phase of the disease, he did not show any signs or symptoms of the disease. He reported having recurrent inflammatory episodes. Adenolymphangitis, i.e., acute inflammatory episodes of the limbs or scrotum that are probably related to bacterial or fungal, secondary infection of tissues with already-compromised lymphatic function, was seen. In the initial phase, he had pitting oedema which later gave rise to brawny oedema with hardening of the tissues. Any malignant illness was ruled out by the absence of any systemic symptoms and the patient's medical history as it was at the time. The patient has no family history or relevant historical experiences. Clinical examination revealed asymmetric swelling of the lower extremities, predominantly affecting the right leg. The skin appeared thickened. Upon palpation, the affected areas appear firm and tender, suggestive of lymphoedema. In addition, there were signs of acute inflammation, with localized warmth and erythema. Following the diagnosis of LF, he is initiated on a comprehensive treatment regimen. In addition to pharmacological treatment, he is counselled on limb care practices, hygiene measures, and strategies to prevent mosquito bites.

## Discussion

Elephantiasis refers to a gradual pathological condition marked by chronic inflammation and enlargement of the connective tissue in the dermis and hypodermis. It is typically preceded by and linked to lymphatic and venous congestion and can result from various factors obstructing or impeding the normal flow of lymphatic and venous fluids [12]. When individuals may not display visible symptoms during this stage, damage to the lymphatic system occurs. This phase can persist for numerous years, during which infected individuals continue to spread the disease. The enduring physical repercussions often manifest as painful swelling in the limbs [13]. Community-wide transmission of the infection can be halted by administering mass treatment with recommended oral regimens of anthelmintic medications such as albendazole, either alone or combined with ivermectin, or diethylcarbamazine citrate in conjunction with albendazole, or a combination of all three, depending on the context. These medications are distributed through mass public health initiatives or, in specific situations, via salt fortification with diethylcarbamazine citrate [14]. The effectiveness of the mass drug administration (MDA) is contingent upon both the efficacy of the treatment regimen and the coverage rate, which represents the proportion of the total population receiving the medication. MDA using two-drug regimens has been successful in breaking the transmission cycle when implemented annually for a minimum of four to six years, ensuring effective coverage across the entire population at risk [15]. India has intensified its endeavours to eradicate LF, a vector-borne illness transmitted by *Culex* mosquitoes, which often results in disability, impacting communities well before global goals, and planned its elimination by the year 2027. The Government of India has proactively introduced a refreshed five-point strategy aimed at eliminating LF, aiming to protect communities from the burdens of disability, social challenges, and economic instability [16]. The circulating filarial antigen (CFA) detection test is now widely considered the definitive method for diagnosing *Wuchereria bancrofti* infections. These assays exhibit nearly complete specificity, and their sensitivity exceeds that of previous parasite-detection techniques [17]. The two pillar strategies for the elimination of LF are annual MDA and home-based management of lymphoedema cases and up-scaling of hydrocele operations in identified community health centers (CHCs)/district hospitals and medical colleges [18]. Collaboration between healthcare providers, government agencies, non-governmental organizations, and local communities is necessary to implement effective control and elimination programs, ultimately reducing the burden of LF on affected populations.

## Conclusions

This case report underscores the importance of early intervention, adherence to treatment, and ongoing support in mitigating the burden of LF and promoting positive outcomes for affected individuals. Moving forward, continued efforts to enhance awareness, access to care, and community-based interventions are essential in the global fight against LF and towards achieving the goal of disease elimination. In addition to emphasizing the significance of prompt diagnosis and comprehensive treatment, this case report underscores the broader implications of LF on public health and socioeconomic development. By addressing the clinical manifestations of LF and its impact on the individual, we also recognize the wider burden placed on healthcare systems, communities, and affected individuals' livelihoods. Furthermore, the importance of a multidisciplinary approach to LF management, involving collaboration between healthcare providers, public health officials, community leaders, and affected individuals themselves, needs to be done. By integrating medical interventions with health education, preventive measures, and social support networks, we can effectively address the multifaceted challenges posed by LF and work towards sustainable disease control and elimination. Ultimately, this serves as a reminder of the urgent need for continued investment, research, and advocacy in the global fight against LF. Through concerted efforts at the local, national, and international levels, we can advance towards a future where LF no longer poses a threat to the health and well-being of communities worldwide.

## References

[1] (2024). Lymphatic filariasis. https://www.who.int/news-room/fact-sheets/detail/lymphatic-filariasis.

[2] (2024). Lymphatic filariasis (elephantiasis). https://www.who.int/health-topics/lymphatic-filariasis.

[3] (2024). Parasites - lymphatic filariasis. https://www.cdc.gov/parasites/lymphaticfilariasis/index.html.

[4] (2024). Lymphatic filariasis - an overview. https://www.sciencedirect.com/topics/medicine-and-dentistry/lymphatic-filariasis.

[5] Lourens GB, Ferrell DK (2019). Lymphatic filariasis. Nurs Clin North Am.

[6] Medeiros ZM, Vieira AV, Xavier AT, Bezerra GS, Lopes MF, Bonfim CV, Aguiar-Santos AM (2021). Lymphatic filariasis: a systematic review on morbidity and its repercussions in countries in the Americas. Int J Environ Res Public Health.

[7] (2024). Lymphatic filariasis (Bancroftian, Malayan, and Timorian). https://publications.aap.org/redbook/book/347/chapter-abstract/5753816/Lymphatic-Filariasis-Bancroftian-Malayan-and.

[8] (2024). Lymphatic filariasis: Progress report 2000-2009 and strategic plan 2010-2020. https://www.who.int/publications/i/item/9789241500722.

[9] Ton TG, Mackenzie C, Molyneux DH (2015). The burden of mental health in lymphatic filariasis. Infect Dis Poverty.

[10] Chavda VP, Pandya A, Pulakkat S, Soniwala M, Patravale V (2021). Lymphatic filariasis vaccine development: neglected for how long?. Expert Rev Vaccines.

[11] Taylor MJ, Hoerauf A, Bockarie M (2010). Lymphatic filariasis and onchocerciasis. Lancet.

[12] Paul E. Simonsen, Gary J. Weil (2024). Elephantiasis. Science direct.

[13] (2024). Lymphatic filariasis (LF). https://ncvbdc.mohfw.gov.in/index4.php?lang=1&level=0&linkid=451&lid=3728.

[14] Ottesen EA, Duke BO, Karam M, Behbehani K (1997). Strategies and tools for the control/elimination of lymphatic filariasis. Bull World Health Organ.

[15] Kumar D, Kumar A, Vikas K, Kumar C, Sircar S (2024). Coverage of mass drug administration (MDA) and operational issues in elimination of lymphatic filariasis in selected districts of Jharkhand, India. J Family Med Prim Care.

[16] (2024). Elimination of lymphatic filariasis. https://ncvbdc.mohfw.gov.in/index4.php?lang=1&level=0&linkid=461&lid=3739.

[17] Agrawal VK, Sashindran VK (2006). Lymphatic filariasis in India : problems, challenges and new initiatives. Med J Armed Forces India.

[18] (2024). Mass drug administration. https://ncvbdc.mohfw.gov.in/index4.php?lang=1&level=0&linkid=462&lid=3740.

